# Gaseous Air Pollution and the Risk for Stroke Admissions: A Case-Crossover Study in Beijing, China

**DOI:** 10.3390/ijerph14020189

**Published:** 2017-02-14

**Authors:** Fangfang Huang, Yanxia Luo, Peng Tan, Qin Xu, Lixin Tao, Jin Guo, Feng Zhang, Xueqin Xie, Xiuhua Guo

**Affiliations:** 1 School of Public Health, Capital Medical University, Beijing 100069, China; hff87hff87@163.com (F.H.); lyx100@ccmu.edu.cn (Y.L.); xuqinhuachong@126.com (Q.X.); taolixin.2008@163.com (L.T.); guojin5827501@163.com (J.G.); zhangfeng@ccmu.edu.cn (F.Z.); 2 Beijing Municipal Key Laboratory of Clinical Epidemiology, Beijing 100069, China; 3 Beijing Public Health Information Center, Beijing 100050, China; tanpeng@bjhb.gov.cn

**Keywords:** stroke, air pollution, hospital admission, exposure-response relationship

## Abstract

*Background:* Though increasing evidence supports association between gaseous air pollution and stroke, it remains unclear whether the effects differ in season, sex and age. The aim of this study was to examine the associations of gaseous air pollution with stroke admissions in Beijing, 2013–2014 in different subgroups. *Methods:* Case-crossover design and conditional logistic regression were used to perform the analyses. We examined the exposure-response relationship between air pollution and stroke. Stratified analyses were performed in different seasons, sex, and age groups. *Results:* There were 147,624 stroke admissions during the study period. In the whole study period, percent changes of stroke admissions were 0.82% (95% CI: 0.52% to 1.13%) and 0.73% (95% CI: 0.44% to 1.03%) per 10 μg/m^3^ increase in the same day conentration of nitrogen dioxide (NO_2_) and sulfur dioxide (SO_2_). The positive associations were higher in warm seasons and with patients >65 years (*p* < 0.05). Contrary effects of carbon monoxide (CO) and ozone on stroke admissions were observed in different seasons. *Conclusions:* NO_2_ and SO_2_ were positively associated with stroke admissions, with stronger effects in warm seasons and with patients >65 years. The associations of CO and ozone with stroke admissions differed across seasons.

## 1. Introduction

Due to increasing motorized traffic and industrial and agricultural activities, concentrations of air pollutants increased substantially in recent decades [1,2,3]. Stroke is a leading cause of death and disability globally, which accounts for five million deaths each year representing nearly 10% of all deaths, and 44 million disability-adjusted life-years are lost annually due to stroke [4]. In China, stroke prevalence estimates in 2013 were statistically greater than those reported in China three decades ago. The age-standardized prevalence, incidence and mortality rates were 1114.8 per 100,000 people, 246.8 and 114.8 per 100,000 person-years, respectively [5]. Therefore, effects of air pollution on stroke should be explored given the great stroke burden in terms of mortality and disability worldwide for primary prevention efforts.

Although exposure to air pollution has been associated with an increase in mortality and morbidity of stroke, these studies were mainly focused on particulate matters (PM) and air pollutants from fossil fuel combustion [6,7,8,9]. For carbon monoxide (CO) and ozone, studies reported inconsistent results [10,11,12,13]. For example, Tian et al. (2015) found that low environmental CO was associated with reduced risk of daily stroke admissions [10] and Männistö et al. (2015) observed decreased odds of stroke events with exposure to ozone [12]. These findings suggest that there may be a non-linear exposure-response relationship which was not well researched. Besides, it remains unclear how air pollution influences stroke in different seasons with varied concentrations.

It is important to understand the characteristics of individuals who are at increased risk of adverse stroke events due to air pollution. Some subpopulations may be particularly sensitive to PM exposure, such as elderly subjects, diabetic patients, and individuals with known coronary artery disease [14,15]. However, prior findings about the modifying effects of characteristics of individuals for gaseous air pollution remain inconsistent. For example, Kan et al. (2008) found that females and the elderly were more vulnerable to nitrogen dioxide (NO_2_), sulfur dioxide (SO_2_), and ozone [16]. Zheng et al. (2013) also found elderly people had higher estimates for cerebrovascular admissions for NO_2_ and SO_2_ than the younger [17]. However, other studies suggest opposite results or no modifying effects of age and sex [8,18].

The aim of this study was to examine the associations of gaseous air pollutants with stroke admissions and their exposure-response relationships, using a case-crossover design, and to determine whether the associations differed in season, sex, and age, in order to capture the susceptible subpopulations.

## 2. Materials and Methods

### 2.1. Air Pollution and Health Data

NO_2_, SO_2_, CO, ozone, and particulate matter that is 2.5 µm or less in diameter (PM_2.5_) measurements in Beijing, China during 1 January 2013 to 31 December 2014 were retrieved from the Centre of City Environmental Protection Monitoring Website Platform of Beijing. The District-level 24-h mean concentrations of air pollutants were used as metrics of exposures. Meteorological data in 16 districts of Beijing were obtained from the Chinese Meteorological Bureau.

We obtained disease data from the medical record database for cardiovascular and cerebrovascular diseases in Beijing. The medical record database contains all the hospitals that have the capability to diagnose and treat cardiovascular and cerebrovascular disease in Beijing. Hospital admissions for stroke (International Classification of Diseases, 10th revision, ICD10: I61-63) and demographic characteristics, including sex and age, between 1 January 2013 to 31 December 2014 were retrieved from the database.

The protocol of this study was approved by the School of Public Health, Capital Medical University (SPHCMU) Institutional Review Board (IRB00009511). Informed consent was not required for this study because all health data were analyzed at the aggregated level, no individual record information for patients was involved and no patients were contacted.

### 2.2. Statistical Analysis

A bidirectional case-crossover design was used to examine the associations of gaseous air pollutants and stroke admissions. The case-crossover design is a variant of the matched case control study and consists of only cases, which serve as their own controls in the analysis. This design can control the effects of day of week, season, and slowly varying confounders. In this study, case period was defined as the day of hospital admission. Control periods were chosen in a two-week window before and after the case period for the same days of the week as the hospital admission [19]. Conditional logistic regression was used to perform the analyses.

Temperature, relative humidity, and holidays were controlled as previous studies have shown that these variables are the potential confounding factors [20,21]. We used natural cubic splines with three degrees of freedom (*df*) to include the effects of temperature and relative humidity on stroke on the day of hospital admission. The public holidays were controlled through binary variable (coded as public holiday = 1 and no public holiday = 0) in the model. All results were from models including temperature, relative humidity, and holidays.

We graphically examined the exposure-response relationship of air pollutants with stroke admissions. We applied natural cubic splines with 3 *df* for NO_2_, SO_2_ and ozone, and 4 *df* for CO to model the relationship. Two-pollutant models adjusting the other gaseous air pollutants were fitted to check the robustness of our results.

Percent change and 95% confidence interval (CI) in stroke admissions associated with a 10.0 μg/m^3^ increase in daily concentration of NO_2_, SO_2_ and ozone, and a 1.0 mg/m^3^ increase in daily concentration of CO were estimated on the same day (lag0), the previous day (lag1), the two preceding days (lag2), and three days moving average (lag0–2).

Stratified analyses were performed to examine whether the associations differed in season (warm season: May to October and cold season: November to April), sex (men and women) and age (>65 years and ≤65 years). Two-pollutant models adjusting PM_2.5_ were fitted to check the modifying effect of PM_2.5_ on associations of gaseous air pollutants and stroke admissions. We tested the statistical significance of subgroup differences through *Z* test [10].

Sensitivity analyses were performed to check the robustness of the results. We used a different method to choose controls, i.e., time-stratified case-crossover design. Degrees of freedom were changed for meteorological variables. We also used temperature and relative humidity lagged by up to two weeks to control the potential lagged effects of meteorological variables.

The conditional logistic regression was performed using the PHREG procedure in SAS statistical software (Version 9.2; SAS Institute, Inc., Cary, NC, USA). We used the Akaike’s Information Criterion to choose the *df* for the splines and determine the goodness of the model fit. All statistical tests were two-sided, and *p*-values less than 0.05 were considered statistically significant.

## 3. Results

There were 147,624 stroke admissions in 2013–2014 that formed the basis for this study. On average there were 205.1 hospital admissions per day for stroke, including 125.7 for men and 119.2 for patients >65 years (Table 1).

Summary statistics of air pollutants and meteorological variables for all days in Beijing, China, 2013–2014 are presented in Table 2. The means (standard deviation, SD) of air pollutants and meteorological variables were 52.5 (28.1) µg/m^3^ for NO_2_, 24.5 (24.8) µg/m^3^ for SO_2_, 1.7 (1.2) mg/m^3^ for CO, 117.5 (73.2) µg/m^3^ for ozone, 89.8 (73.2) µg/m^3^ for PM_2.5_, 11.9 (11.2) °C for temperature, and 56.1 (17.5) % for relative humidity, respectively. On warm days, the means (SD) of NO_2_, SO_2_, CO, and ozone were 44.9 (21.5) µg/m^3^, 11.4 (11.0) µg/m^3^, 1.2 (0.6) mg/m^3^, and 163.7 (69.1) µg/m^3^, respectively. On cold days, the corresponding values were 60.1 (31.2) µg/m^3^, 43.7 (38.7) µg/m^3^, 2.2 (1.4) mg/m^3^, and 70.6 (39.9) µg/m^3^, respectively.

Spearman correlation coefficients for correlations among the exposure variables are presented in Table 3. NO_2_, SO_2_, and CO were positively correlated with each other (correlation coefficient, *r* = 0.58 to 0.68, *p* < 0.001) and negatively correlated with ozone (*r* = −0.43 to –0.29, *p* < 0.001). Correlations between temperature and NO_2_, SO_2_, and CO were negative (*r* = −0.29 to –0.42, *p* < 0.001). Correlation between temperature and ozone was positive (*r* = 0.79, *p* < 0.001).

**Figure 1:**
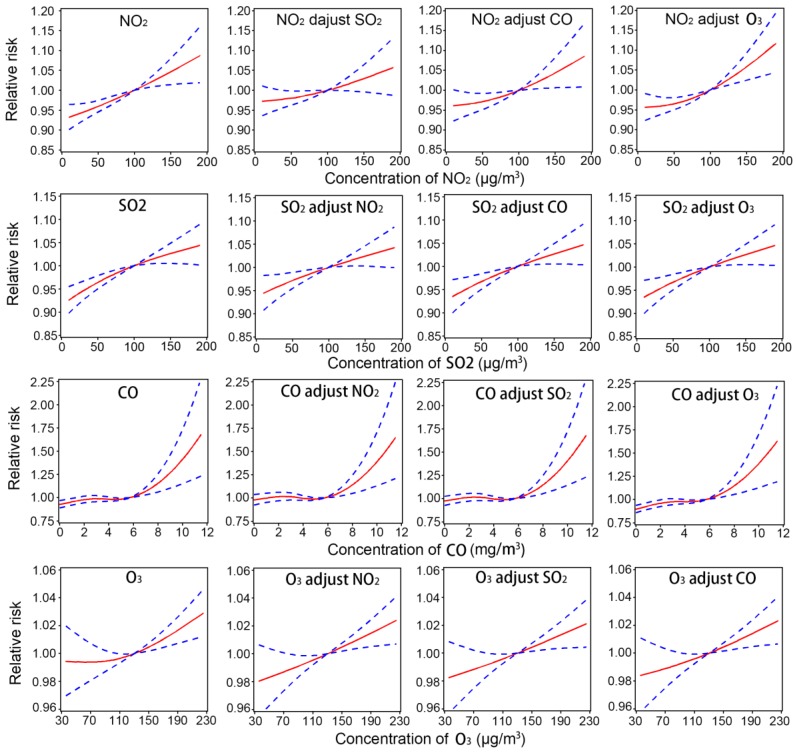
Exposure-response relationships of stroke admissions with air pollutants on the concurrent day in different pollutant models.

Exposure-response relationships of stroke admissions with air pollutants in different pollutant models are shown in Figure 1. The exposure-response curves suggest an approximately linear rise in stroke risk with daily changes in NO_2_, SO_2_, and ozone concentrations in both single- and two-pollutant models. For CO, there was a linear increase in stroke risk at higher CO concentrations, with a threshold of around 5 mg/m^3^ for a 24-h average exposure.

Percent changes (95% CI) in hospital admissions for stroke associated with a 10.0 μg/m^3^ increase in NO_2_, SO_2_, and ozone, and a 1.0 mg/m^3^ increase in CO are shown in Table 4.

In the whole study period, NO_2_ was positively associated with stroke admissions on the same day (0.82%, 95% CI: 0.52% to 1.13%) and the previous day (0.39%, 95% CI: 0.11% to 0.67%). When stratified by season, stroke admissions were positively associated with the same day, previous day, and three days moving average concentration of NO_2_ in warm season (*p* < 0.05). However, in cold seasons, the positive association was only observed on the same day (0.40%, 95% CI: 0.02% to 0.78%). The associations were higher in warm days than in cold days at lag0 (*Z* = 5.426, *p* < 0.001), lag1 (*Z* = 5.735, *p* < 0.001), and lag0–2 day (*Z* = 5.516, *p* < 0.001).

Similar with NO_2_, SO_2_ was positively associated with stroke admissions at lag0 (0.73%, 95% CI: 0.44% to 1.03%) and lag1 (0.40%, 95% CI: 0.11% to 0.68%) day in the whole study period. In both warm and cold seasons, positive associations with SO_2_ were observed on the same day and the previous day (*p* < 0.05), with statistically significant higher values in warm seasons at lag0 day (*Z* = 3.406, *p* = 0.001).

For CO, associations with stroke admissions were different when stratified by seasons. In cold seasons, stroke admissions were positively associated with the same day (2.47%, 95% CI: 1.54% to 3.41%) and the previous day (1.04%, 95% CI: 0.25% to 1.84%) concentration of CO. However, in warm seasons, CO was negatively associated with stroke admissions at lag1 (−4.26%, 95% CI: −5.98% to −2.51%), lag2 (−5.87%, 95% CI: −7.51% to −4.20%) and lag0–2 (−7.95%, 95% CI: −10.34% to −5.49%) day.

In addition, a 10 µg/m^3^ increase in the same day and the previous day concentration of ozone corresponded to a 0.23% (95% CI: 0.08% to 0.37%) and 0.22% (95% CI: 0.09% to 0.36%) increase in stroke admissions in the whole study period. However, the associations were different when stratified by season. The ambient ozone was positively associated with stroke admissions in warm season, and negatively associated with stroke admissions in cold season (*p* < 0.05).

**Figure 2:**
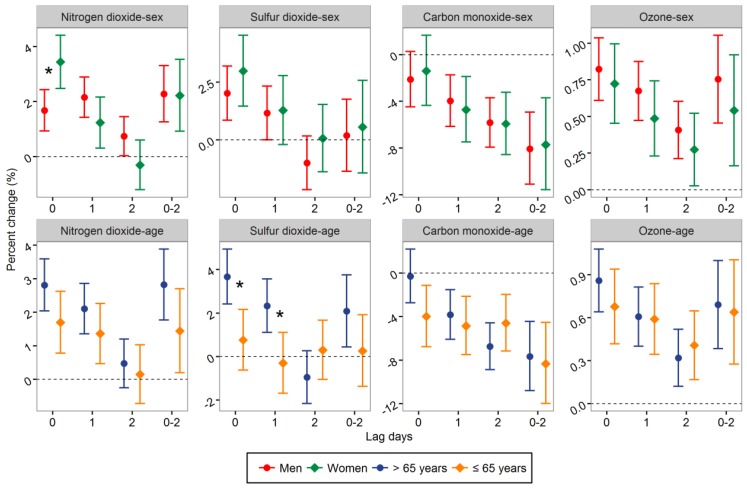
Percent change (95% CI) in hospital admissions for stroke associated with a 10.0 μg/m^3^ increase in NO_2_, SO_2_, and ozone, and 1.0 mg/m^3^ increase in CO stratified by sex and age in warm seasons. * The difference between two subgroups was statistically significant (*p* < 0.05).

Percent changes in stroke admissions stratified by sex and age were calculated in different seasons. In warm seasons, positive associations of stroke admissions with NO_2_ and SO_2_ appeared to be stronger in patients >65 years, with statistically significant higher values for patients >65 years at lag0 (*Z* = 3.021, *p* = 0.003) and lag1 day (*Z* = 2.766, *p* = 0.006) for SO_2_ (Figure 2). The positive association of stroke admissions with the same day concentration of NO_2_ was significantly higher in female patients (*Z* = −2.830, *p* = 0.005). However, the results were inconsistent in the other lag structures.

**Figure 3:**
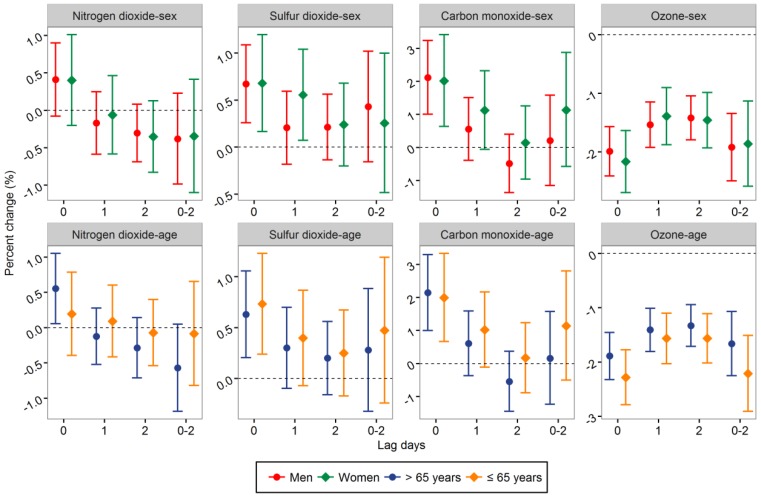
Percent change (95% CI) in hospital admissions for stroke associated with a 10.0 μg/m^3^ increase in NO_2_, SO_2_, and ozone, and 1.0 mg/m^3^ increase in CO stratified by sex and age in cold seasons.

No statistically significant differences were found in different sex and age subgroups in cold seasons (Figure 3).

Two-pollutant models adjusting PM_2.5_ were performed in both warm and cold seasons (Table 5 and Table 6). In warm seasons, the positive associations of stroke admissions with NO_2_, SO_2_, and ozone were weaker in two-pollutant model than in single-pollutant model. The differences were statistically significant for NO_2_ at lag1 (*Z* = 2.530, *p* = 0.011) and lag2 day (*Z* = 2.078, *p* = 0.038). The negative associations of stroke admissions with CO became stronger after adjusting PM_2.5_. The differences were statistically significant at lag0 (*Z* = 2.738, *p* = 0.006) and lag0–2 day (*Z* = 2.194, *p* = 0.028).

In cold seasons, two-pollutant models show that adjustment for PM_2.5_ had no obvious impact on the associations of stroke admissions with NO_2_, SO_2_, and ozone. The positive association of stroke admissions with CO at lag0 day was higher after adjusting PM_2.5_ (*Z* = −2.520, *p* = 0.012).

Sensitivity analyses show that changing the method of choosing controls, the degrees of freedom and the lag days of temperature and relative humidity did not change the main findings (Table 7).

## 4. Discussion

This study suggests an approximately linear exposure-response relationship of stroke with NO_2_ and SO_2_, and they were positively associated with stroke admissions. The results were consistent with previous findings worldwide and supported by epidemiological and experimental studies [8,18,22,23,24]. The putative biological mechanisms linking air pollution to cardiovascular diseases involve direct effects on the cardiovascular system, blood, and lung receptors, and/or indirect effects mediated through pulmonary oxidative stress and inflammatory responses [23,24,25].

Some studies examined the seasonal difference in effects of NO_2_ and SO_2_ on stroke but generated inconsistent results [8,17,26]. For example, Wichmann and Voyi (2012) reported positive associations of cerebrovascular mortality with NO_2_ in warm season [8], whereas Xiang et al. (2013) observed opposite results [26]. We found that the positive associations of NO_2_ and SO_2_ with stroke admissions were higher in warm seasons. Combined effects of high air pollution and temperature levels, and the varied ventilation conditions across seasons may explain the seasonal difference [6].

The subgroup analyses show that the positive associations of stroke admissions with NO_2_ and SO_2_ appearing to be stronger in patients >65 years in warm seasons, consistent with previous studies [16,17]. The evidence for interaction between air pollution and comorbid factors on stroke risk is growing [27,28]. Potentially, preexisting respiratory or cardiovascular conditions are more prevalent for the elderly than the younger. The vulnerable condition in the elderly could impact the effects of air pollutants on stroke [29]. Another possible theory behind the difference is that recurrent stroke is more common for older patients. Previous studies suggest that short-term trigger effects of air pollution on cardiovascular events are factors primarily for patients with a history of cardiovascular disease [30,31]. Furthermore, the proportion of stroke subtype may be different between two age groups, and the association of air pollution with stroke may differ across stroke subtype [32]. Thus, analyses including information on types of previous stroke diseases and comorbid factors might help to understand potential pathways in future studies.

There were two recent meta-analyses examining the acute effects of air pollution on stroke, and both reported a significantly positive association between ambient CO exposure and the same day stroke hospitalizations [33,34]. However, Tian et al. (2015) found that low environmental CO was associated with reduced risk of daily stroke hospitalizations [10]. In this study, an almost linear exposure-response relationship of stroke admissions at higher concentrations of CO was observed with a threshold value.

When stratified by season, negative associations between stroke admissions and CO in warm season with lower CO levels were observed. These findings suggest that ambient CO at low levels may have no or beneficial effects on stroke. Furthermore, the anti-inflammatory and beneficial neuroprotective effects of CO under certain circumstances have been suggested by recent experimental studies [35,36]. Therefore, the short-term beneficial effects of low environmental CO against stroke are biologically plausible.

Although several epidemiological studies have examined the association of ambient ozone with stroke, they reported inconsistent results. Some studies reported statistically significant and positive associations [37,38], whereas others presented no or negative associations [12,39,40]. This study found that exposure to ambient ozone was positively associated with stroke admissions in warm season, but negatively associated with stroke admissions in cold season, similar with the results reported by Villeneuve et al. (2012) [41].

Some studies stated that the negative association of ozone with stroke might be attributed to its negative association with other air pollutants, which were more strongly associated with cardiovascular events [12]. Furthermore, one meta-analysis reported a positive association between stroke and ambient ozone exposure only in a subgroup of Asia area [34]. They stated that the observed geographic differences of the associations may reflect a shape difference of exposure-response function for stroke between low and high pollution settings. In this study, daily mean concentration of ozone was lower in cold season. The seasonal difference may be attributed to the difference in ozone levels. Notably, ozone was highly correlated with temperature in this study. This relationship together with a misspecified temperature model might also contribute to the difference between seasons.

Previous studies suggest that PM_2.5_ may increase the risk of stroke admissions and mortality [6,7]. It is necessary to check the modification effects of PM_2.5_ on the associations of gaseous pollutants and stroke admissions. Two-pollutant models show that PM_2.5_ had no obvious impact on the associations of stroke admissions with SO_2_ and ozone [17,42]. For NO_2_, the effects diminished after adjustment for PM_2.5_ in warm season [43]. However, the directions of effect estimates did not change. Therefore, effects of NO_2_, SO_2_, and ozone on stroke admissions in single-pollutant models were reliable. In this study, the negative associations of CO with stroke admissions in warm season and positive associations in cold season both became stronger [44]. The effects of CO on stroke may be underestimated.

There are several strengths of this study. First, the effect estimates in case-crossover design are probably not due to confounding by age, gender, smoking, underlying chronic disease, or other individual-level characteristics. Moreover, the medical record database contains all hospitals with the capability to diagnose and treat cardiovascular and cerebrovascular disease in Beijing and provides a large number of cases. District-daily-level air pollutants and the large range of concentrations used in this study can provide better control of measurement error than use of data from one monitoring site and small range of concentrations.

This study has several potential limitations. First, possible misclassification of diseases may exist related to the unrecognized occurrence of stroke and non-hospitalized individuals, with the onset of stroke symptoms in the days prior to hospital admissions. Furthermore, the use of district-level concentrations rather than individual exposure may lead to measurement error. Given the ecological design of this study, caution should be exercised in inferring cause-effect relations, especially for ozone, which is possibly non-linearly correlated with stroke.

## 5. Conclusions

NO_2_ and SO_2_ were positively associated with stroke admissions, with stronger effects in warm seasons, and in patients >65 years. This study also suggests an approximately linear exposure-response relationship of stroke admissions at higher CO levels, with a threshold effect. The associations of CO and ozone with stroke admissions differed by season.

## Figures and Tables

**Table 1:** Summary statistics of stroke admissions in Beijing, China, 2013–2014.

Count/Day	Mean	SD	Median	IQR	Minimum	Maximum
All days						
All	205.1	62.8	212.0	113.0	81.0	375.0
Men	125.7	40.1	128.0	70.0	42.0	225.0
Women	79.4	24.5	82.0	42.0	28.0	150.0
Age > 65	119.2	37.9	126.0	69.0	41.0	215.0
Age ≤ 65	85.8	26.7	85.0	43.0	27.0	179.0
Warm days						
All	206.6	64.4	210.0	118.5	81.0	375.0
Men	127.2	41.5	129.0	75.0	51.0	225.0
Women	79.4	24.7	81.5	42.5	28.0	150.0
Age > 65	120.5	39.5	126.5	72.0	45.0	208.0
Age ≤ 65	86.1	26.7	86.0	44.5	36.0	179.0
Cold days						
All	203.6	61.2	213.5	108.0	81.0	340.0
Men	124.1	38.6	128.0	65.0	42.0	218.0
Women	79.5	24.4	82.5	41.0	30.0	134.0
Age > 65	118.0	36.3	126.0	64.0	41.0	215.0
Age ≤ 65	85.6	26.8	85.0	43.0	27.0	152.0

SD, standard deviation; IQR, interquartile range.

**Table 2:** Summary statistics of air pollutants and meteorological variables in Beijing, China, 2013–2014.

Variables	Mean	SD	Med	IQR	Min	Max
All days						
NO_2_ (μg/m^3^)	52.5	28.1	48.3	36.3	1.0	196.8
SO_2_ (μg/m^3^)	24.5	24.8	14.8	27.1	1.0	197.1
CO (mg/m^3^)	1.7	1.2	1.3	1.0	0.0	11.5
Ozone (μg/m^3^)	117.5	73.2	99.0	102.5	0.0	703.0
PM_2__.5_ (μg/m^3^)	89.8	73.2	71.4	82.0	4.0	685.5
Temperature (°C)	11.9	11.2	13.5	20.7	−13.3	31.1
Relative humidity (%)	56.1	17.5	56.5	27.2	12.8	96.0
Warm season						
NO_2_ (μg/m^3^)	44.9	21.5	42.4	58.0	1.0	159.9
SO_2_ (μg/m^3^)	11.4	11.0	8.0	14.0	1.0	197.1
CO (mg/m^3^)	1.2	0.6	1.0	1.5	0.0	9.0
Ozone (μg/m^3^)	163.7	69.1	160.0	101.1	2.0	703.0
PM_2__.5_ (μg/m^3^)	79.8	57.7	66.6	70.3	4.0	373.0
Temperature (°C)	20.9	5.5	22.0	7.7	5.0	31.1
Relative humidity (%)	64.1	15.2	65.3	21.1	18.4	94.3
Cold season						
NO_2_ (μg/m^3^)	60.1	31.2	55.7	79.0	1.0	196.8
SO_2_ (μg/m^3^)	43.7	38.7	34.0	57.5	2.0	537.0
CO (mg/m^3^)	2.2	1.4	2.0	3.0	0.1	11.5
Ozone (μg/m^3^)	70.6	39.9	67.0	44.2	0.0	331.0
PM_2__.5_ (μg/m^3^)	100.6	84.8	80.0	92.9	4.3	685.5
Temperature (°C)	2.5	7.1	1.3	10.5	−13.3	20.9
Relative humidity (%)	47.8	15.7	46.8	22.0	12.8	96.0

SD, standard deviation; Med, median; IQR, interquartile range; Min, minimum value; Max, maximum value; NO_2_, nitrogen dioxide; SO_2_, sulfur dioxide; CO, carbon monoxide; PM_2.5_, particulate matter that is 2.5 µm or less in diameter.

**Table 3:** Spearman correlation coefficients among the exposure variables.

Variable	NO_2_	SO_2_	CO	Ozone	PM_2.5_	T	RH
NO_2_	1.00	0.58 *	0.61 *	−0.29 *	0.61 *	−0.24 *	0.13 *
SO_2_	-	1.00	0.68 *	−0.46 *	0.48 *	−0.60 *	−0.21 *
CO	-	-	1.00	−0.43 *	0.68 *	−0.42 *	0.18 *
Ozone	-	-	-	1.00	−0.09 *	0.79 *	0.15 *
PM_2.5_	-	-	-	-	1.00	−0.05 *	0.40 *
T	-	-	-	-	-	1.00	0.36 *
RH	-	-	-	-	-	-	1.00

NO_2_, nitrogen dioxide; SO_2_, sulfur dioxide; CO, carbon monoxide; PM_2.5_, particulate matter that is 2.5 µm or less in diameter; T, temperature; RH, relative humidity. * *p* < 0.001.

**Table 4:** Percent change (95% CI) in hospital admissions for stroke associated with a 10.0 μg/m^3^ increase in NO_2_, SO_2_, and ozone, and 1.0 mg/m^3^ increase in CO stratified by season.

Variables	All Days	Season		Test for Difference between Seasons	
		Warm Season	Cold Season	*Z* Value	*p* Value
NO_2_					
Lag0	0.82 (0.52 to 1.13) ^#^	2.35 (1.76 to 2.94) ^#^	0.40 (0.02 to 0.78) *	5.426	<0.001
Lag1	0.39 (0.11 to 0.67) *	1.80 (1.22 to 2.37) ^#^	−0.13 (−0.46 to 0.20)	5.735	<0.001
Lag2	−0.14 (−0.40 to 0.12)	0.34 (−0.22 to 0.90)	−0.08 (−0.38 to 0.23)	1.281	0.200
3 days average	0.22 (−0.18 to 0.61)	2.24 (1.44 to 3.05) ^#^	−0.37 (−0.84 to 0.10)	5.516	<0.001
SO_2_					
Lag0	0.73 (0.44 to 1.03) ^#^	2.38 (1.45 to 3.32) ^#^	0.67 (0.35 to 1.00) ^#^	3.406	0.001
Lag1	0.40 (0.11 to 0.68) *	1.20 (0.28 to 2.12) *	0.34 (0.04 to 0.64) *	1.741	0.082
Lag2	0.16 (−0.10 to 0.42)	0.43 (−0.45 to 1.33)	0.22 (−0.05 to 0.49)	0.451	0.652
3 days average	0.39 (−0.03 to 0.81)	0.32 (−0.91 to 1.56)	0.36 (−0.10 to 0.82)	−0.065	0.948
CO					
Lag0	1.35 (0.60 to 2.11) ^#^	−1.86 (−3.70 to 0.01)	2.07 (1.20 to 2.95) ^#^	−3.714	<0.001
Lag1	0.34 (−0.33 to 1.01)	−4.26 (−5.98 to −2.51) ^#^	0.78 (0.04 to 1.52) *	−5.143	<0.001
Lag2	−0.40 (−1.03 to 0.23)	−5.87 (−7.51 to −4.20) ^#^	−0.25 (−0.94 to 0.45)	−6.016	<0.001
3 days average	−0.01 (−0.95 to 0.95)	−7.95 (−10.34 to −5.49) ^#^	0.56 (−0.50 to 1.64)	−6.103	<0.001
Ozone					
Lag0	0.23 (0.08 to 0.37) *	0.78 (0.62 to 0.95) ^#^	−2.06 (−2.39 to −1.73) ^#^	14.946	<0.001
Lag1	0.22 (0.09 to 0.36) *	0.60 (0.44 to 0.76) ^#^	−1.48 (−1.78 to −1.18) ^#^	11.862	<0.001
Lag2	0.03 (−0.11 to 0.16)	0.36 (0.20 to 0.51) ^#^	−1.44 (−1.73 to −1.15) ^#^	10.556	<0.001
3 days average	0.20 (0.00 to 0.40)	0.67 (0.44 to 0.91) ^#^	−1.90 (−2.35 to −1.45) ^#^	9.833	<0.001

CI, confidence interval; NO_2_, nitrogen dioxide; SO_2_, sulfur dioxide; CO, carbon monoxide. * *p* < 0.05; **^#^** *p* < 0.001.

**Table 5:** Percent change (95% CI) in stroke admissions associated with a 10.0 μg/m^3^ increase in NO_2_, SO_2_, and ozone, and 1.0 mg/m^3^ increase in CO after adjusting PM_2.5_ in warm season.

Variables	Single-Pollutant Model	Model Adjusting PM_2.5_	*Z* Value *	*p* Value *
NO_2_				
Lag0	2.35 (1.76 to 2.94)	1.63 (0.97 to 2.30)	1.572	0.116
Lag1	1.80 (1.22 to 2.37)	0.70 (0.08 to 1.33)	2.530	0.011
Lag2	0.34 (−0.22 to 0.90)	−0.51 (−1.08 to 0.06)	2.078	0.038
3 days average	2.24 (1.44 to 3.05)	1.07 (0.17 to 1.97)	1.907	0.056
SO_2_				
Lag0	2.38 (1.45 to 3.32)	1.91 (0.89 to 2.93)	0.666	0.505
Lag1	1.20 (0.28 to 2.12)	0.56 (−0.39 to 1.51)	0.946	0.344
Lag2	0.43 (−0.45 to 1.33)	−3.04 (−3.98 to −2.09)	0.866	0.386
3 days average	0.32 (−0.91 to 1.56)	−1.00 (−2.28 to 0.30)	1.438	0.150
CO				
Lag0	−1.86 (−3.70 to 0.01)	−5.68 (−7.65 to −3.66)	2.738	0.006
Lag1	−4.26 (−5.98 to −2.51)	−6.73 (−8.48 to −4.96)	1.957	0.050
Lag2	−5.87 (−7.51 to −4.20)	−5.90 (−7.57 to −4.21)	0.028	0.978
3 days average	−7.95 (−10.34 to −5.49)	−11.79 (−14.19 to −9.32)	2.194	0.028
Ozone				
Lag0	0.78 (0.62 to 0.95)	0.71 (0.53 to 0.89)	0.573	0.567
Lag1	0.60 (0.44 to 0.76)	0.41 (0.24 to 0.58)	1.631	0.103
Lag2	0.36 (0.20 to 0.51)	0.21 (0.06 to 0.37)	1.257	0.209
3 days average	0.67 (0.44 to 0.91)	0.42 (0.17 to 0.67)	1.444	0.149

CI, confidence interval; NO_2_, nitrogen dioxide; SO_2_, sulfur dioxide; CO, carbon monoxide; PM_2.5_, particulate matter that is 2.5 µm or less in diameter. * Test for difference between models.

**Table 6:** Percent change (95% CI) in stroke admissions associated with a 10.0 μg/m^3^ increase in NO_2_, SO_2_, and ozone, and 1.0 mg/m^3^ increase in CO after adjusting PM_2.5_ in cold seasons.

Variables	Single-Pollutant Model	Model Adjusting PM_2.5_	*Z* Value *	*p* Value *
NO_2_				
Lag0	0.40 (0.02 to 0.78)	0.72 (0.12 to 1.31)	−0.870	0.385
Lag1	−0.13 (−0.46 to 0.20)	−0.23 (−0.60 to 0.14)	0.407	0.684
Lag2	−0.08 (−0.38 to 0.23)	−0.38 (−0.69 to −0.06)	0.232	0.816
3 days average	−0.37 (−0.84 to 0.10)	−0.65 (−1.23 to −0.07)	0.738	0.460
SO_2_				
Lag0	0.67 (0.35 to 1.00)	0.76 (0.39 to 1.12)	−0.336	0.737
Lag1	0.34 (0.04 to 0.64)	0.39 (0.05 to 0.74)	−0.224	0.823
Lag2	0.22 (−0.05 to 0.49)	0.03 (−0.26 to 0.33)	0.913	0.361
3 days average	0.36 (−0.10 to 0.82)	0.38 (−0.12 to 0.89)	−0.072	0.943
CO				
Lag0	2.07 (1.20 to 2.95)	4.13 (2.79 to 5.49)	−2.520	0.012
Lag1	0.78 (0.04 to 1.52)	0.69 (−0.11 to 1.51)	0.146	0.884
Lag2	−0.25 (−0.94 to 0.45)	−0.62 (−1.33 to 0.10)	0.736	0.461
3 days average	0.56 (−0.50 to 1.64)	0.77 (−0.50 to 2.05)	−0.242	0.809
Ozone				
Lag0	−2.06 (−2.39 to −1.73)	−2.23 (−2.57 to −1.90)	0.732	0.464
Lag1	−1.48 (−1.78 to −1.18)	−1.54 (−1.85 to −1.22)	0.265	0.791
Lag2	−1.44 (−1.73 to −1.15)	−1.32 (−1.63 to −1.02)	−0.547	0.584
3 days average	−1.90 (−2.35 to −1.45)	−2.12 (−2.58 to −1.65)	0.652	0.514

CI, confidence interval; NO_2_, nitrogen dioxide; SO_2_, sulfur dioxide; CO, carbon monoxide; PM_2.5_, particulate matter that is 2.5 µm or less in diameter. * Test for difference between models.

**Table 7:** Percent change (95% CI) in stroke admissions associated with a 10.0 μg/m^3^ increase in NO_2_, SO_2_, and ozone, and 1.0 mg/m^3^ increase in CO in sensitivity analyses.

Variables	Sensitivity Analysis I *	Sensitivity Analysis II ^#^	Sensitivity Analysis III ^†^
NO_2_			
Lag0	0.77 (0.45 to 1.09)	0.82 (0.52 to 1.13)	1.14 (0.87 to 1.40)
Lag1	0.38 (0.10 to 0.66)	0.39 (0.11 to 0.67)	0.75 (0.49 to 1.02)
Lag2	−0.17 (−0.44 to 0.10)	−0.14 (−0.41 to 0.12)	0.08 (−0.19 to 0.34)
3 days average	0.42 (0.02 to 0.83)	0.20 (−0.20 to 0.60)	0.91 (0.56 to 1.27)
SO_2_			
Lag0	0.38 (0.07 to 0.69)	0.72 (0.43 to 1.02)	1.11 (0.83 to 1.39)
Lag1	0.20 (−0.09 to 0.50)	0.40 (0.11 to 0.68)	0.76 (0.49 to 1.04)
Lag2	−0.18 (−0.45 to 0.09)	0.16 (−0.10 to 0.42)	0.35 (0.08 to 0.61)
3 days average	−0.02 (−0.46 to 0.43)	0.36 (−0.06 to 0.78)	1.11 (0.70 to 1.51)
CO			
Lag0	1.19 (0.42 to 1.98)	1.32 (0.56 to 2.10)	2.36 (1.68 to 3.04)
Lag1	0.41 (−0.28 to 1.10)	0.35 (−0.33 to 1.03)	1.33 (0.65 to 2.01)
Lag2	−0.25 (−0.90 to 0.40)	−0.43 (−1.07 to 0.22)	−0.09 (−0.75 to 0.58)
3 days average	0.43 (−0.55 to 1.43)	−0.13 (−1.10 to 0.84)	2.01 (1.07 to 2.95)
Ozone			
Lag0	0.29 (0.14 to 0.44)	0.24 (0.09 to 0.38)	0.23 (0.10 to 0.36)
Lag1	0.16 (0.02 to 0.30)	0.23 (0.09 to 0.37)	0.24 (0.11 to 0.37)
Lag2	−0.07 (−0.21 to 0.07)	0.03 (−0.10 to 0.17)	0.07 (−0.06 to 0.20)
3 days average	0.28 (0.08 to 0.49)	0.22 (0.02 to 0.42)	0.12 (−0.06 to 0.31)

CI, confidence interval; NO_2_, nitrogen dioxide; SO_2_, sulfur dioxide; CO, carbon monoxide. * Using time-stratified case-crossover design. **^#^** Changing the temperature and relative humidity degrees of freedom to four instead of three. **^†^** Adjusting temperature and relative humidity lagged by up to two weeks.
